# Cytoprotective Effect of* Ligustrum robustum* Polyphenol Extract against Hydrogen Peroxide-Induced Oxidative Stress via Nrf2 Signaling Pathway in Caco-2 Cells

**DOI:** 10.1155/2019/5026458

**Published:** 2019-06-13

**Authors:** Jiayi Chen, Fangting He, Sijing Liu, Tao Zhou, Saira Baloch, Chunping Jiang, Xiaofang Pei

**Affiliations:** West China School of Public Health and West China Fourth Hospital, Sichuan University, 16#, Section 3, Renmin Road South, Chengdu 610041, Sichuan, China

## Abstract

*Ligustrum robustum* is a traditional herbal tea that is widely distributed in southwest China. The health effects of* L. robustum* are characteristics of clearing heat, antioxidant, inducing resurgence, and improving digestion. However, the molecular mechanisms related to these effects, particularly the antioxidant mechanism, have been seldom reported. The objective of this study was to assess antioxidative capacity of* L. robustum*, and its protective effects and mechanisms against hydrogen peroxide (H_2_O_2_) - induced toxicity in Caco-2 cells. Total phenolic contents, free radical scavenging activity, and reducing capacity of* L. robustum* were measured. The effects of* L. robustum* on the cell viability and antioxidant defense system were explored. The expression of nuclear factor E2 related factor 2 (Nrf2) and antioxidant genes: quinone oxidoreductase 1 (NQO1), heme oxygenase-1 (HO-1), and glutamate cysteine ligase (GCL) were analyzed by western blot and qPCR. Pretreatment of* L. robustum* could significantly reduce H_2_O_2_-induced toxicity, decrease the level of reactive oxygen species (ROS) and malondialdehyde (MDA), and increase the activities of superoxide dismutase (SOD), catalase (CAT), glutathione peroxidase (GSH-Px), and glutathione reductase (GR). By activating the expression of Nrf2 and antioxidant genes (NQO1, HO-1, and GCL),* L. robustum* exerts cytoprotective effect in Caco-2 cells dealt with H_2_O_2_. Therefore, the well-established model of Caco-2 cells demonstrates that* L. robustum* may modulate the cytoprotective effect against the H_2_O_2_-induced oxidative stress through the Nrf2 signaling pathway.

## 1. Introduction

Oxidative stress (OS) refers to the disequilibrium in the production and degradation of reactive oxygen species (ROS). ROS are generated in electron transport chain and play an important role in metabolisms [1]. However, excessive production of ROS may damage biomolecules and lead to the pathological changes, such as inflammation, cancer, neurodegeneration, cardiovascular diseases, and inflammatory bowel disease (IBD) [2, 3].

To neutralize OS, cells have evolved adaptive mechanisms. Activation of antioxidant defense systems could benefit cells survival [4, 5]. Nuclear factor-erythroid-2-related factor 2 (Nrf2) is one of the main transcription factors mediating cellular defense [4, 5]. Nrf2 is quiescent and restricted by Kelch-like ECH-associated protein 1 (Keap1) under basal conditions. In response to stimuli, Nrf2 will activate, translocate into the nucleus, and bind to antioxidant response element (ARE) [6]. Thus, Nrf2-ARE initiates cellular defense by induction of phase II detoxifying and antioxidant enzymes, i.e., quinone oxidoreductase 1 (NQO1), heme oxygenase-1 (HO-1), superoxide dismutase (SOD), and catalase (CAT) [6].

A number of dietary polyphenols have been reported to modulate Nrf2 pathways [7–10]. Natural plants have received much attention due to low toxicity, high antioxidant efficiency, and protective effect against oxidative damage. Kuding Cha, traditional tea substitute, has acted as a functional herb in China for over two thousand years.* Ligustrum robustum* belongs to Oleaceae family, which is the main original plant of Kuding Cha. According to ancient record, its leaves were consumed as herbal tea for clearing heat, detoxification, to refresh the mind and sedate, improve digestion, and to boost memory. Moreover,* L. robustum* was classified as a food by the Chinese Ministry of Health [11]. Recent studies have revealed that* L. robustum* holds potential effects on obesity [12], influenza [13], and inflammatory responses [14]. Furthermore,* L. robustum* extracts showed protective effects against hydrogen peroxide (H_2_O_2_) - induced cytotoxicity as previously studied [15]. However, the molecular mechanisms involved in antioxidant activity are unclear. Whether Nrf2 pathway is contributed to the antioxidant properties of* L. robustum* needs further research.

Therefore, the objective of the current study was to characterize the anti-oxidative capacity of* L. robustum* and its protective effects against H_2_O_2_ treated cells and to provide evidence for possible mechanisms. Human colon adenocarcinoma cells (Caco-2) represent well-established, reliable cell line which has been utilized to evaluate antioxidant properties of natural extracts. OS of the cells was determined by cellular ROS, malondialdehyde (MDA), glutathione (GSH) contents, and activities of antioxidant enzymes. Nrf2/ARE pathway activation and expression of associated antioxidant enzymes were also demonstrated.

## 2. Methods

### 2.1. Chemicals and Materials

6-Hydroxy-2,5,7,8-tetramethylchroman-2-carboxylic acid (Trolox), 2,2-diphenyl-1-picrylhydrazyl (DPPH), Folin-Ciocalteu reagents, chlorogenic acid, ferrozine, hydrogen peroxide (H_2_O_2_), 2,2′-Azobis (2-methylpropionamidine) dihydrochloride (AAPH), 2,7-dichlorofluorescein diacetate (DCFH-DA), and fluorescein sodium were purchased from Sigma Chemical Co. (St. Louis, Missouri, United States). Other solvents and chemicals of analytical grade were commercially available. The leaves of* Ligustrum robustum *were purchased from the largest* L. robustum* provider in China, Qing Shan Lu Shui Co., Ltd. (Junlian, Sichuan, China).

Cell Counting Kit-8 (CCK-8) was obtained from Dojindo Molecular Technologies, Inc. (Kumamoto, Japan). The measurement kits for malondialdehyde (MDA), reduced glutathione (GSH), and superoxide dismutase (SOD) were obtained from Nanjing Jiancheng Bioengineering Institute (Nanjing, China). And other kits for total antioxidative capacity (ABTS methods), Ferric reducing antioxidative potential (FRAP), BCA (bicinchoninic acid) protein assay, catalase (CAT), glutathione peroxidase (GSH-Px), and glutathione reductase (GR) were obtained from Beyotime Biotechnology (Shanghai, China). RNAprep Pure Cell Kit was purchased from Tiangen Biotechnology (Beijing, China). cDNA Synthesis Kit and SYBR Green Super mix were acquired from Biorad (California, USA). All other cell culture chemicals and reagents were obtained from HyClone (Logan, UT, USA) and Gibco BRL, Life Technologies (USA).

### 2.2. Preparation of Plant Extracts

The leaves of* Ligustrum robustum *(50 g) were extracted with redistilled water (500 ml) at 80°C in a water bath for 1 h for three times. The extract of* L. robustum *was filtered, combined, and concentrated (60°C) in rotary evaporator to acquire the crude extract. Then the crude extract was suspended in redistilled water and fractioned with chloroform, ethyl acetate and n-butanol by separating funnel. The fractioned extracts were then concentrated* in vacuo* and dissolved in redistilled water.

### 2.3. Determination of Total Polyphenols Content

Polyphenols contents were determined by Folin-Ciocalteu procedure [16]. Concisely 1 ml of the plant extract was mixed with 3 ml of Folin-Ciocalteu reagent and incubated at room temperature for 5 min. Then 4.5 ml of 7.5% Na_2_CO_3_ solution was added to the mixture, which was incubated at room temperature for 90 min in the dark. The absorbance of the mixture was measured at 747 nm by Microplate Reader Multiskan GO, Thermo Fisher Scientific (Waltham, Massachusetts, United States) and the total phenolic content was expressed as mg of chlorogenic acid equivalents (CAE)/g of dried plant material.

### 2.4. Antioxidant Assays

#### 2.4.1. ABTS Radical Scavenging Assay

The ABTS assay was carried out based on the method of Total Antioxidant Capacity Assay Kit with ABTS method. 400 *μ*l potassium persulfate was added to 400 *μ*l ABTS to generate ABTS stock solution, which was incubated at room temperature in the dark for 16 h. The stock solution was diluted with phosphate buffer before use and the absorbance was adjusted to 0.70 ± 0.05 at 734 nm (ABTS working solution). Various extracts (10 *μ*l) were added to ABTS working solution (200 *μ*l) at room temperature. After 6 min of reaction the absorbance was measured at 734 nm. The antioxidant activities of these extracts were displayed by trolox equivalents antioxidant capacity as mmol trolox equivalents/g extract.

#### 2.4.2. DPPH Radical Scavenging Activity

DPPH radical scavenging activity was carried out with slight modifications [17]. 0.1 ml of various concentrations of extract was added to 2 ml of DPPH (6.25×10^−5^ M) solution in ethanol and the mixture was incubated in the dark at room temperature for 30 min. The absorbance of remaining solutions was measured at 517 nm. DPPH radical scavenging activity was displayed as mmol trolox equivalents (TE)/g of dried plant material.

#### 2.4.3. Oxygen Radical Absorption Capacity Assay

Oxygen radical absorption capacity (ORAC) was measured by fluorescence quenching [18, 19]. In brief, a range of standards in phosphate buffer and fluorescein sodium (40 nM) were applied to a 96-well black plate. The plates were incubated at 37°C for 10 min and then the AAPH was added to the mixture. The fluorescence measurements were continuously taken for 60 times at excitation of 485 nm and emission of 527 nm by EnVision Multilabel Reader, Perkin Elmer (Waltham, Massachusetts, United States). ORAC value was expressed as *μ*mol trolox/g of dried plant material.

#### 2.4.4. Ferric Reducing Antioxidative Potential (FRAP)

FRAP was determined according to Total Antioxidant Capacity Assay Kit with FRAP method. Acetate buffer, tripyridyltriazine (TPTZ) and ferric chloride (10:1:1) were mixed to form FRAP working solution at 37°C for 30min and the absorbance of the mixed solution was read at 450nm. FRAP value was expressed as mmol trolox equivalents/g of dried plant material.

#### 2.4.5. Cell Culture and Viability

Human colon adenocarcinoma, Caco-2 cells were maintained in antibiotic-free Dulbecco's modified Eagle's medium (DMEM) supplemented with 10% fetal bovine serum, 100 units/ml penicillin, and 100 *μ*g/ml streptomycin at 37°C in a humidified atmosphere of 5% CO_2_. Caco-2 cells were cultured in 96-wells plate at a density of 2× 10^4^ cells/well for 48h. To assess the cytotoxicity of extracts, cells were incubated with n-butanol extracts (BuE) at 37°C for 24 h. To assess the protective effects, cells were pretreated with BuE for 24 h and then were washed by PBS for three times. Then the cells were treated by 200 *μ*mol/l H_2_O_2_ for 2 h at 37°C to induce OS. Cell viability was determined by CCK-8 and optical density was read at 450 nm by microplate reader. The untreated cells were the blank group, and the cells treated with hydrogen peroxide only were the control or model group. Cell viability was expressed as percentage relative to blank group. The cells were processed, washed, and centrifuged for further tests.

#### 2.4.6. Determination of Cellular ROS

The production of intracellular ROS was measured by 2,7-dichlorofluorescein diacetate (DCFH-DA) [20]. After 24 h incubation of BuE, 10 *μ*mol/l DCFH-DA was added to the wells for 30 min. Cells were washed by PBS for three times and were treated with 200 *μ*M H_2_O_2_ for 2 h. The fluorescence intensity was determined at excitation of 488nm and emission of 525nm using EnVision Multilabel Reader.

#### 2.4.7. Analyses for Total Antioxidant Capacity, MDA, GSH, and Antioxidant Enzymes

Total antioxidant capacity of cell lysates, GSH, MDA, and antioxidant enzymes (SOD, GSH-Px, CAT, GR) activities were performed using the commercial assay kits. Detailed operations were performed according to the manufacturer's instructions. Protein levels of the cell samples were measured using the BCA method according to the manufacturer's instructions.

#### 2.4.8. mRNA Expression of Nrf2 and Related Genes Determined by RT-PCR

Total RNA was extracted by RNAprep Pure Cell Kit (Tiangen, Beijing, China) according to the manufacturer's instructions. The cDNA was synthesized by cDNA Synthesis Kit (Biorad, California, USA). The primers (Table 1) were synthesized from Life Technology Corp (Shanghai, China). 1 *μ*l specific primers (10 nM), 2× PCR SYBR Green Super mix (Biorad, California, USA) and 1 *μ*l of cDNA were added to a total volume of 20 *μ*l reaction. PCR was conducted on the CFX 96 thermocycler (Biorad, California, USA) under the conditions, i.e., initial denaturation at 95°C for 1 min, denaturation at 95°C for 5s, annealing, and extension at 58-60°C for 5s for 40 amplification cycles. *β*-actin was used for normalization.

### 2.5. Western Blot Analysis

After treatments, cells were lysed with RIPA lysis buffer on ice. The lysates were scraped from the plates and centrifuged at 12000 g for 5 min. Total protein concentrations were measured by BCA method. Equal amounts of protein were separated by 10% SDS polyacrylamide gel electrophoresis and electrotransferred to PVDF membranes (Millipore). The membranes were blocked with TBS containing 5% nonfat milk for 1 h and were immunoblotted at 4°C overnight with primary antibodies (Servicebio technology, Wuhan, China, 1:1000) for Nrf2 or *β*-actin. The membranes were washed with TBS (containing 20% Tween) three times and were incubated with horseradish peroxidase-conjugated secondary antibodies (Servicebio technology, Wuhan, China, 1:3000) for 1h at room temperature. Bands were visualized by the chemiluminescence method (Bio-Rad Laboratories).

### 2.6. Statistical Analysis

The experiment was performed in triplicate and repeated three times. Data were expressed as the mean ± standard deviation (SD). One-way ANOVA, Dunnett's, and SNK test were performed using SPSS 18.0 version. Differences were considered significant when p values were below 0.05 (*p*<0.05).

## 3. Results

### 3.1. Total Phenolic Contents and Antioxidant Capacities of* L. rotustum* Extracts

The contents of total phenolic were in the following order (Table 2): n-butanol fraction > ethyl acetate fraction > water fraction > chloroform fraction. Phenolic contents and antioxidant capacities varied a lot with different extraction solvents. The ability of scavenging DPPH radical, ABTS radical, and absorbing oxygen radical and reducing ferric ion were as follows: n-butanol fraction > ethyl acetate fraction > water fraction > chloroform fraction. n-butanol extract showed the highest total phenolic contents and antioxidant capacities.

### 3.2. Cytoprotective Effect of* L. rotustum* Extracts on H_2_O_2_-Induced Cell Damage

**Figure 1:**
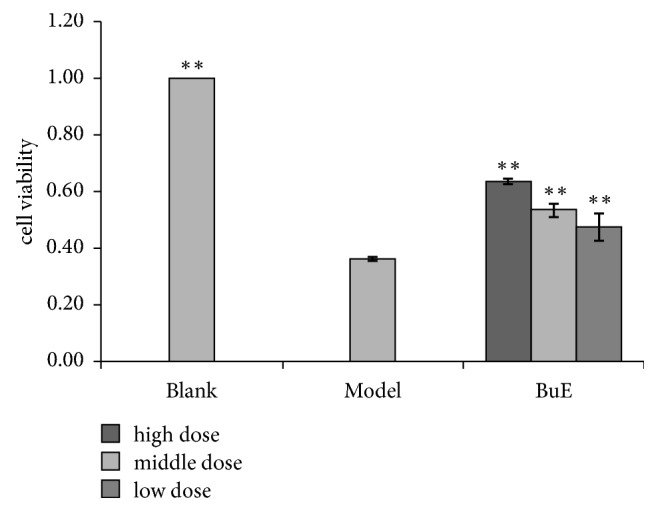
Effects of and BuE on cell viability in H_2_O_2_-injuried Caco-2 cell. Caco-2 cells were pretreated with extracts for 24h prior to H_2_O_2_ for 2h. After the treatment, cell viability is determined by CCK-8 analysis. Data are shown as means ± SD (n = 6). *∗∗*p < 0.01 versus model. BuE: n-butanol extract.

Decreasing the concentrations of BuE improved cell viability. BuE was nontoxic to Caco-2 cells for 24 h up to a concentration of 162.5 mg (chlorogenic acid)/l. When exposed to H_2_O_2_ (model group), the viability of Caco-2 cells, which was only 36.2%, decreased compared to control cells, whereas the pretreated with BuE (162.5mg/l, 81.3mg/l, and 40.6mg/l), cell viability increased by 75.3%, 47.8%, and 31.2% in comparison to the model group (Figure 1). BuE significantly abrogated H_2_O_2_-induced cytotoxicity in a dose-dependent manner. These findings demonstrated that BuE of* L. robustum *has significant cytoprotective effects on H_2_O_2_-induced cytotoxicity in Caco-2 cells.

### 3.3. Intracellular ROS Generation

**Figure 2:**
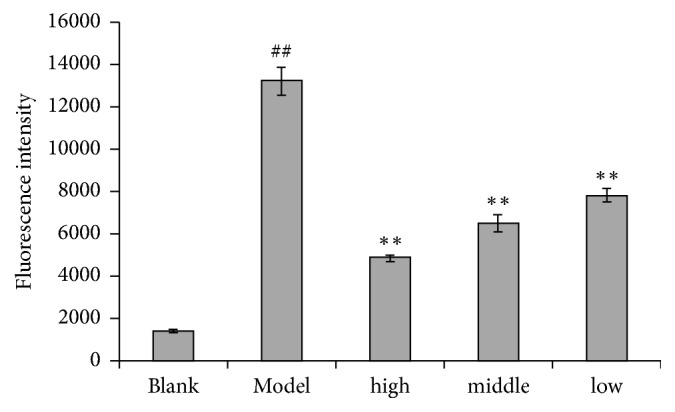
Effects of BuE with high, middle and low concentrations on ROS levels in Caco-2 cells. Caco-2 cells were pretreated with BuE for 24 h prior to H_2_O_2_ for 2 h. ##p < 0.01 versus blank. *∗∗*p < 0.01 versus model.

ROS production was measured by DAFH-DA assay. ROS generation of the model group was significantly increased up to tenfold as compared to the control group (Figure 2). ROS level was significantly decreased when pretreated with BuE of different concentrations compared to the model group.

### 3.4. MDA, GSH, Total Antioxidant Capacity, and Antioxidant Enzyme Activities

Lipid peroxidation was determined by the formation of malondialdehyde (MDA) in the homogenates of Caco-2 cells. MDA concentration in the model group was significantly elevated nearly 130.8% compared to the control group, while BuE inhibited MDA level. In high and middle dose group MDA contents decreased to 60.1% and 32.3%, respectively, in comparison with the model group. No significant difference was found between low dose group and the model group (Table 3). GSH concentration was decreased by 44.5% compared to the control group. Pretreatment with BuE has ability to prevent GSH depletion compared to the model group. Especially the high dose group prevented GSH depletion as near as the control group.

In order to find out whether the protective effect was dependent on the antioxidant functions, the activities of antioxidant enzymes were measured. The activities of SOD in the model group increased by 28.8% compared to the control group. The activities of CAT, GSH-Px, and total antioxidant capacity (TAOC) in the model group decreased by 36.2%, 28.1%, and 45.6%, respectively, compared to the control group. The activity of GR was not significantly different with the control group. In groups pretreated with BuE, SOD, CAT, GSH-Px, GR, and TAOC activity significantly increased compared with the model group. Besides, SOD, CAT, and TAOC in middle dose group showed the highest activities and GSH-Px, GR showed the highest activities in high dose group.

### 3.5. Expressions of Nrf2 and Antioxidant Enzymes in Caco-2 Cells

**Figure 3:**
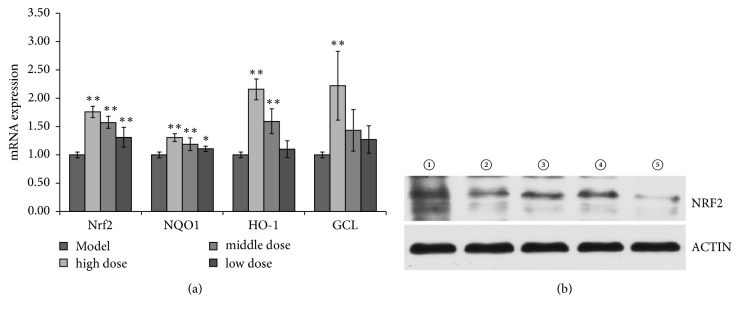
Effects of BuE with various concentrations on the expressions of antioxidant enzymes and Nrf2 in Caco-2 cells. (a) Gene expressions of Nrf2, NQO1, HO-1, and GCL. Data are presented as mean ± SD, *∗*p < 0.05, *∗∗*p < 0.01 versus model. (b) The levels of Nrf2 were analyzed by Western blot. 1: blank group, 2: model group, 3: high dose, 4: middle dose, and 5: low dose. The test was repeated three times and representative blots are shown.

In this context, we observed that Nrf2, NQO1, HO-1, GCL, SOD, and CAT mRNA expression were significantly decreased upon H_2_O_2_ treatment in comparison to untreated cells. However, pretreatment of the cells with BuE (high and medium dose) markedly increased mRNA expression compared to H_2_O_2_-treated cells (Figure 3).

Western blotting analysis exhibited the presence of a 96 kDa band corresponding to the Nrf2 molecular weight, which showed lighter band after H_2_O_2_ stimulation compared with the control. When high and medium dose were added to Caco-2 cells, the Nrf2 levels were significantly increased in comparison to the levels obtained in H_2_O_2_-treated cells. Overall, these data suggested that* L. robustum* can upregulate Nrf2 expression at the transcription level and may further activate downstream antioxidant proteins in cellular antioxidant response.

## 4. Discussion

ROS are byproducts of aerobic metabolisms, participating in energy metabolism and host defense [21, 22]. H_2_O_2_ is major sources of ROS and directly damages the biomolecules through production of hydroxyl radicals [23]. Gastrointestinal (GI) tract is a key reservoir of ROS [24] and OS is considered as one of the etiologic factors involved in a variety of GI disorders including IBD, GI malignancies, and gastroduodenal ulcers [24]. Various studies suggest that natural compounds exert their protective and therapeutic effect on GI disorders through numerous molecular mechanisms, including antioxidative stress and modulation of intracellular signaling transduction pathways [25]. Phenolic compounds are secondary metabolites in plants and important sources of human diet with many health benefits [26, 27]. Kuding Cha polyphenols have long been thought to have many bioactivities [28]. However, previous studies mostly focused on large leaf Kuding Cha (Ilex Kuding Cha). Bioactivities and molecular mechanisms of small leaf Kuding Cha polyphenols, such as* L. robustum*, have not been extensively discussed.

Caco-2 cells are important intestinal cell types and are used for evaluations of antioxidant capacity of food and natural extracts [29]. We employed H_2_O_2_-induced OS in Caco-2 cells, to investigate the antioxidant capacity and possible mechanisms of* L. robustum*, to provide a scientific basis for the development and utilization of antioxidant new product against GI disorders. In this study, mechanisms concerning antioxidative effects of* L. robustum* may be accompanied by scavenging ROS and activating Nrf2 expression to improve endogenous antioxidant defenses has been shown.

Pretreatment with BuE of* L. robustum* increased cell viability, decreased ROS level, and improved SOD, CAT, GSH-Px, and GR activities compared to the H_2_O_2_ group, indicating BuE significantly attenuated H_2_O_2_-induced cell damage through reducing ROS level and maintaining endogenous antioxidant enzymes. However, middle dosage group displayed the highest SOD, CAT, and TAOC activities. It could be attributed that the high dosage extracts may exert antioxidant activities by directly scavenging free radicals, while the medium dosage may mostly rely on the expression of antioxidant enzymes, such as SOD and CAT. Above all, middle dosage group may display the highest TAOC activities.

Many reports have indicated that phytochemicals could induce the expression of phase II enzyme genes. Nrf2 is a basic leucine zipper transcription factor, sensitive to oxidative and electrophilic stress [30]. Nrf2 pathway plays a crucial role in the control of detoxifying enzymes, antiapoptotic proteins, and proteasomes [31]. NQO1 is a flavoprotein enzyme that promotes the reductions of quinones, nitroaromatics, and azo dyes [32, 33]. HO-1 could protect against oxidative stress-induced cytotoxicity and is thought to be beneficial to GI diseases including gastritis, peptic ulcers, and esophagitis [34, 35]. Glutamate cysteine ligase (GCL) is major determinant and rate-limiting enzyme of GSH synthesis [36].

In the present study, we have found that pretreatment with BuE significantly upregulated mRNA expression of Nrf2, NQO1, HO-1, and GCL in a concentration-dependent manner and the expression of Nrf2 were higher in middle dosage group cell than the model group. However, Nrf2 expressions were not observed in low dose group. It has been reported that low and moderate dose H_2_O_2_ may accumulate Nrf2 in the nuclear and upregulate ARE-medicated gene expression, while high dose H_2_O_2_ led to nuclear exclusion of Nrf2 [37, 38]. In this study, low doses of BuE may directly interact with ROS and there was not enough extract left to activate Nrf2. Considering the regulation of H_2_O_2_ on Nrf2, the expression Nrf2 of low dosage group may be weaker than the control group, suggesting that cytoprotective effect of this group may largely depend on ROS scavenging activities of* L. robustum*.

Collectively, present study investigated the antioxidant capacity of polyphenol extracts of* L. robustum *and its protective effect against H_2_O_2_-induced oxidative damage in Caco-2 cells. Results showed that* L. robustum* could reduce cellular ROS and improve the activities of antioxidant enzymes. In addition, the expressions of antioxidant enzymes may be attributed to the activation of Nrf2. It could conclude that cytoprotective effect of* L. robustum* depends on scavenging ROS and modulation of endogenous antioxidant systems. The n-butanol fraction of* L. robustum* could be considered as a potential natural antioxidant and a novel inducer of phase II antioxidant enzymes and Nrf2.

Further research needs to be carried out on the pathway by which* L. robustum* may activate the translocation of Nrf2. Besides Nrf2, whether other redox-associated transcription factors, such as AP-1 and NF-*κ*B, were involved in the effects of* L. robustum* on the regulation of antioxidant enzymes requires further research. It is also needed to separate and identify bioactive constituents responsible for the antioxidant activity, to explore its effects on the prevention and treatment of the oxidative stress associated with human diseases.

## Figures and Tables

**Table 1:** Primers used for the amplification of Nrf2 and related genes.

Gene	Primer Sequences	Fragment	Annealing	Accession Number
Nrf2	F: AGT GGA TCT GCC AAC TAC TC	105bp	60°C	NM001313904.1
	R: CAT CTA CAA ACG GGA ATG TCT G			
NQO1	F: CGC AGA CCT TGT GAT ATT CCA GT	87bp	60°C	NM001025433.1
	R: TCC TAT GAA CAC TCG CTC AAA CC			
HO-1	F: CCA GGC AGA GAA TGC TGA GT	156bp	58°C	NM002133.2
	R: GTA GAC AGG GGC GAA GAC TG			
GCL	F: ATG GAG GTG CAA TTA ACA GAC	204bp	58°C	XM017010749.1
	R: CTG CAT TGC CAC CTT TGC A			
*β*-actin	F: GGC ACC CAG CAC AAT GAA	160bp	58°C	NM001101.3
	R: CTA AGT CAT AGT CCG CCT AGA AGC A			

**Table 2:** Total polyphenolic contents and antioxidant capacities of various extracts of *Ligustrum robustum*.

	Total phenol contents	ABTS	ORAC	DPPH	FRAP
	mg CAE/g dried leaves	mmol Trolox/g	*μ*mol Trolox/g	mmol Trolox/g	mmol/g
Chloroform	1.20±0.01^abc^	0.0088±0.0002^abc^	8.50±0.70^abc^	0.0010±0.0001^abc^	0.0074±0.0008^abc^
Ethyl acetate	29.77±0.11^a^	0.2342±0.0153^a^	236.40±31.63^a^	0.0806±0.0029^a^	0.1865±0.0224^a^
n-butanol	75.70±0.29	0.4496±0.0104	420.75±49.42	0.2634±0.0052	0.5102±0.0408
Water	9.78±0.06^ab^	0.0662±0.0029^ab^	40.42±3.93^ab^	0.0276±0.0006^ab^	0.0684±0.0055^ab^

The values reported are mean ± SD. CAE: chlorogenic acid (a: different from n-butanol fraction, b: different from ethyl acetate fraction, c: different from remaining water fraction, *p*<0.05).

**Table 3:** Effects of BuE with various doses on the marker of oxidative stress in Caco-2 cells.

	Blank	H_2_O_2_	BuE + H_2_O_2_		
			162.5 mg/l	81.3 mg/l	40.6 mg/l
MDA (nmol/g protein)	0.36±0.15	0.82±0.19##	0.33±0.11*∗∗*	0.56±0.16*∗∗*	0.64±0.11
GSH (*μ*mol/g protein)	51.52±2.85	28.59±4.58##	52.21±3.94*∗∗*	43.65±0.82*∗∗*	39.95±2.30*∗∗*
TAOC (mmol /g protein)	0.41±0.01	0.22±0.01##	0.31±0.01*∗∗*	0.34±0.01*∗∗*	0.28±0.01*∗∗*
SOD (U/mg protein)	21.48±1.86	27.66±1.46##	30.47±1.99*∗∗*	36.93±2.16*∗∗*	33.76±1.05*∗∗*
CAT (U/mg protein)	26.20±0.23	16.72±0.30##	22.18±0.98*∗∗*	29.83±1.03*∗∗*	25.11±0.83*∗∗*
GSH-Px (mU/mg protein)	3.75±0.23	2.70±0.36##	4.87±0.32*∗∗*	4.15±0.27*∗∗*	3.69±0.31*∗∗*
GR (mU/mg protein)	3.14±0.43	3.36±0.14	4.59±0.21*∗*	3.83±0.12*∗*	3.63±0.28

Data are shown as means ± SD (n = 6). BuE: n-butanol extract. ##p < 0.01 versus blank. *∗*p < 0.05 versus H_2_O_2_. *∗∗*p < 0.01 versus H_2_O_2_

## Data Availability

The data used to support the findings of this study are available from the corresponding author upon request.
